# A Longitudinal Study on Dual-Tasking Effects on Gait: Cognitive Change Predicts Gait Variance in the Elderly

**DOI:** 10.1371/journal.pone.0099436

**Published:** 2014-06-06

**Authors:** Rebecca K. MacAulay, Robert M. Brouillette, Heather C. Foil, Annadora J. Bruce-Keller, Jeffrey N. Keller

**Affiliations:** 1 Louisiana State University, Department of Psychology. Baton Rouge, Louisiana, United States of America; 2 Pennington Biomedical Research Center, Baton Rouge, Louisiana, United States of America; Oregon Health & Science University, United States of America

## Abstract

Neuropsychological abilities have found to explain a large proportion of variance in objective measures of walking gait that predict both dementia and falling within the elderly. However, to this date there has been little research on the interplay between changes in these neuropsychological processes and walking gait overtime. To our knowledge, the present study is the first to investigate intra-individual changes in neurocognitive test performance and gait step time at two-time points across a one-year span. Neuropsychological test scores from 440 elderly individuals deemed cognitively normal at Year One were analyzed via repeated measures t-tests to assess for decline in cognitive performance at Year Two. 34 of these 440 individuals neuropsychological test performance significantly declined at Year Two; whereas the “non-decliners” displayed improved memory, working memory, attention/processing speed test performance. Neuropsychological test scores were also submitted to factor analysis at both time points for data reduction purposes and to assess the factor stability overtime. Results at Year One yielded a three-factor solution: Language/Memory, Executive Attention/Processing Speed, and Working Memory. Year Two's test scores also generated a three-factor solution (Working Memory, Language/Executive Attention/Processing Speed, and Memory). Notably, language measures loaded on Executive Attention/Processing Speed rather than on the Memory factor at Year Two. Hierarchal multiple regression revealed that both Executive Attention/Processing Speed and sex significantly predicted variance in dual task step time at both time points. Remarkably, in the “decliners”, the magnitude of the contribution of the neuropsychological characteristics to gait variance significantly increased at Year Two. In summary, this study provides longitudinal evidence of the dynamic relationship between intra-individual cognitive change and its influence on dual task gait step time. These results also indicate that the failure to show improved test performance (particularly, on memory tests) with repeated administrations might prove to be useful of indicator of early cognitive decline.

## Introduction

Understanding factors associated with individual differences in gait within the elderly is an important direction for research, given that abnormal gait patterns have been found to be a risk marker for dementia, as well as being predictive of falls in the elderly. Research suggests that neurocognitive abilities play an important role in individual differences in walking gait patterns. In particular, executive functioning and attention related processes appear to explain a large proportion of variance in certain objective clinical measures of gait that predict falling within the elderly [1], [2], [3], [4], [5]. However, to this date there has been little research on the interplay between neuropsychological processes and walking gait, and how changes in cognition contribute to gait variance overtime. To address this issue, this study examined neuropsychological characteristics that appear to be the most sensitive to change in the elderly, and their influence on dual task step time at two distinct time points spaced approximately a year apart from one another within a relatively healthy elderly sample.

The neuropsychological processes most affected by age-related decline are what have been commonly termed: executive functioning, attention, processing speed, episodic memory, and language abilities [6]. Executive functioning, which has an inhibitory influence on attention and working memory processes, in particular appears to be compromised in elderly who exhibit early signs of mild cognitive impairment (MCI) [7], [8], [9]. A common characteristic amongst these factor is frontal lobe involvement, in which age related changes within this region appear to impact the capacity to plan, initiate, and execute goal-directed behaviors (see [10], [11]). In addition to these neuropsychological processes being sensitive to age-related decline, impairments in these processes are believed to play an important role in the relationship between abnormal walking gait patterns and poor dual-task performance within elderly populations. Specifically, there is evidence that age related decline in executive attention processes might underlie the effect of dual-task performance decrement on walking gait parameters [4], [11], [12], [13], [14]. However, while age related cognitive decline is posited to be an important predictor of gait change in the elderly, to our knowledge no study has examined the relationship between cognitive change and dual task gait performance overtime within the elderly. Additionally, given posited age related shifts in cognitive resources and potential compensatory mechanisms (e.g., [7], [15]) questions remain regarding the stability of certain neurocognitive factor structures within the elderly. In this regard, the present study adds to the body of existing literature through providing longitudinal data on the effect of intra-individual variability in neurocognitive performance and its relationship with dual-task gait step time performance.

The effect of *dual-* as compared *single* task conditions upon performance has been well documented. There is a wide body of research that suggests that simultaneously performing two tasks divides attentional resources, which consequently results in some form of performance decrement [16]. While dual task conditions widely vary, the present study's primary focus is on dual tasks that involve the participant performing a cognitive task while doing a motor activity (e.g., asking a participant to spell a word backward aloud as they are walking). Additionally, while dual task decrements (DTD; defined as the difference between gait measure in time or length within single as compared to dual task condition) are found within healthy young adults, the “cost” of dual task conditions upon walking gait appears to substantially increase with age and has been linked to impairment in neuropsychological functioning (for review, see [11]). Furthermore, older adults appear to require greater attentional resources than younger adults do to perform a dual-task [17]. Here, it is important to note, that the level of difficulty of the cognitive task component to the dual task also appears to play an important role in DTD [18]. For example, a study that explored the effect of forward as compared to backward counting on gait stride time found that backward counting and not forward counting produced greater gait variance in elderly individuals with frontal lobe dysfunction [19]. Furthermore, careful review of the literature suggests that investigating objective measures of gait speed under dual-task conditions that test mental tracking (i.e., the ability to follow a sequence of events) or working memory will be more likely to differentiate between groups of healthy participants and those with neurological deficits (see [20]). In summary, there appears to be a clear relationship between neuropsychological functioning and DTD in gait patterns within the elderly.

Research indicates that language abilities, executive attention, processing speed, and memory functioning are all predictive of variance in gait velocity during single task conditions; however, only executive functioning, attention/processing speed and memory factors appear to predict changes in gait velocity during dual task conditions within relatively healthy elderly participants (aged 70 or older) [14]. Poor executive functioning in particular appears to potentiate the relationship between poor dual-task performance and cognitive–motor tasks within the elderly [12], [13]. Likewise, in elderly adults with dementia, gait time variability during dual task conditions appears to be impacted by impaired executive control over attentional processes. Specifically, poor attention allocation appears to be responsible for the observed increased time and variability in stride time during dual-task conditions [19]. Furthermore, decline in executive functioning, and not memory functioning, has been found to mediate the relationship between dual-task and abnormal gait performance in elderly who are prone to falls but not in “non-fallers” [21]. Overall, research suggests that executive functioning and/or attention substantially influence gait and its stability.

There are several parameters used in the measurement of gait abnormalities, *step time* in particular appears to be sensitive to changes in cognition and the effect of DTD, as it appears to require recruitment of cortical control [22]. Importantly, within individuals with MCI significant increases in step time and its variance, but not gait velocity or stride length, have been found during the dual- as compared to the single-task condition [23]. In this regard, it appears that the variable step time might be more impacted by dual tasks than other gait parameters, and that individual differences in cognitive functioning would be expected to explain a substantial amount of variance in gait step time in dual task conditions.

Cognitive functioning clearly appears to play an important role in the relationship between DTD and gait patterns within the elderly. The present study investigated changes in neurocognitive performance at two-time points across a one-year span in order to examine the effect of cognitive changes on gait step time during a dual task condition. First, we examined neuropsychological characteristics that have been posited to be sensitive to change in the elderly (e.g., [6]). Groups were then formed based on whether there was a statistically significant decline in neurocognitive test performance (“decliners” vs. “non-decliners”) at Year Two. Neuropsychological test scores were also submitted to principal components factor analyses for data reduction purposes; as well as, we were interested in investigating whether there was variance in the factor structure between Years One and Two. Based on prior research [24], we hypothesized that a four-factor model consisting of: memory, attention/processing speed, executive function, and language would best fit the data; and that this would be relatively invariant across the two time points. Lastly, regression analyses were employed within each group to investigate the unique contributions of individual differences in neuropsychological and demographics factors in explaining variance in dual task gait step time at Years One and Two. We hypothesized that individual differences in executive functioning and attention/processing speed would contribute to variance in gait step time during a dual task amongst those that displayed cognitive decline as well as the non-cognitive decline group at both time points; however, we predicted that the contribution of executive attention and processing speed to step time variance would increase over time in the cognitive decline group.

## Materials and Methods

### Participants

Participants are part of an on-going longitudinal study (Louisiana Aging Brain Study: LABrainS) that investigates the effects of aging upon cognitive processes and daily living functioning. Volunteers for the LABrainS study are recruited for the study through regular outreach efforts of the Institute for Dementia Research and Prevention (IDRP) throughout Louisiana. Eligibility criteria for this study requires that participants be willing to undergo annual cognitive assessment and are over the age of 60 with no existing diagnosis of dementia or cognitive impairment at the time of baseline screening. All participants have a Clinical Dementia Rating of 0 and a Mini-Mental Status Exam (MMSE) [25] score >25, consistent with the absence of dementia. Exclusion criteria for LABrainS includes: a Geriatric Depression Scale score ≥6 (15 item version) [26], a history of neurological or untreated health conditions (e.g., cerebrovascular disease, Parkinson's disease, and/or a traumatic brain injury, etc.) that might cause cognitive sequelae. Oral and written informed consent was obtained from participants at each clinic visit and the study was approved by the Pennington Biomedical Institutional Review Board and Ethics Committee.

In the present study all participants were deemed cognitively normal for age at Year One [as defined by a Uniform Data Set (UDS) score of 2; see below for detail]. A total of 440 participants who had completed objective cognitive assessment measures in both 2011 and 2012 were eligible to participate. At Year Two, two groups: “non-decliners” vs “decliners” were formed based on whether there was a statistically significant decline in individuals' neuropsychological test performance (defined by changes in their UDS score from a 2 to 3). Table 1 presents demographic variables by group. Overall, the proportion of females to males (65.5% female vs. 34.5% male) was higher within the sample. The mean years of education was 16.16 years (*SD*  =  2.35), and the mean age was approximately 70 years old (*SD*  =  7.67). Of these 440 participants, 400 completed objective gait assessment (non-decliners *n* = 370 and decliners *n* = 30).

**Table 1:** Demographic Variables by Group.

	Non-decliner	Decliner
Sex (% female)	68	59
Education (years)	16.15 (2.35)	16.24 (2.46)
Age (years)	70.28 (6.91)	73.29 (7.37)

### Neuropsychological Assessment

The UDS Neuropsychological battery established by the National Alzheimer's Coordinating Center (NACC) was utilized for this study. The NACC test battery consists of brief measures of attention, processing speed, executive function, episodic memory, and language that were selected due to their sensitivity to detect cognitive change in the elderly [6]. The UDS specific tests include: a measure of dementia severity (MMSE), Wechsler's Memory Scale-Revised (WMS-R) [27] Logical Memory IA and IIA, WMS-R Digit Span Forward and Backward, Category Fluency (animals and vegetables) [28], Trails Making Test (TMT): Parts A and B [29], Wechsler's Adult Intelligence Scale- Revised Digit Symbol Subtest [30], and the 30 odd-numbered items of the Boston Naming Test (BNT) [28]. In addition to the UDS neuropsychological test battery, the Clock Drawing Test (CDT) was administered as measure of a spatial reasoning and memory. UDS appraisal scores are based on whether individuals' neuropsychological performance is above (superior test performance  =  1), normal (all tests scores falling within normal range  =  2), slightly abnormal (one or two abnormal test scores  =  3), or clinically impaired (≥ 3 abormal test scores  =  4) for age.

### Gait Assessment

The GAITRite system (CIR Systems, Inc., Sparta, NJ) provides an objective, valid and reliable measurement of walking gait [31]. The GAITRite is an electronic carpeted walkway (overall dimensions: 90 cm×700 cm×3.2 mm) with encased pressure sensors within the mat that allow for collection of information regarding the respective components that make up an individual's walking gait (e.g., step time, cadence, and step width). This study specifically focused on step time, defined as the time elapsed from first contact of one foot to first contact of the opposite foot in seconds, as its primary measure of gait. A step time average score for both single task and dual task condition trials was created for each individual.

## Study Procedures

The study's procedures were conducted by well trained, certified research assistants. For each year, participants first underwent informed consent followed by neuropsychological testing in a private testing suite. Subsequently, GAITRite is administered in an adjacent well lit hallway for a total of four trials (2 per condition with single task trials always being administered before the dual task trials). For the single task trials, participants were instructed to walk across the walkway “using their normal everyday walking speed”. For the dual task condition (the third and fourth trials), participants were instructed to walk across the mat just like before, except for this time they would be asked to spell a word backwards aloud as they walked. All words were five letters in length and of equal difficulty (for word list, see GAITRite manual).

## Analyses

Demographic variables were evaluated to assess for their potential influence at all levels of analyses. Descriptive statistics were computed for all variables to determine that assumptions of normality (skewed scores: | ≤ 1|) and homogeneity of variance were met. Three outliers (defined as greater than 2.5 standard deviations from the mean) data were removed in order to correct for skew in step time in the non-decliner group, and analyses were re-run without these skewed values. Cognitive test scores were standardized relative to the mean of the entire sample. Two-tailed tests were used to compute all *p*-values.

Analyses were conducted in three phases. First, group differences in demographic and neuropsychological variables were examined using chi-square tests or One-Way ANOVAs. Repeated measure t-tests examined within group differences in neuropsychological test performance at Year One compared to Year Two. Second, for data reduction purposes, neuropsychological test scores were submitted to principal components factor analysis with eigenvalues set above 1. Orthogonal and oblique rotation factor matrices were compared and presented similar factor solutions. Data presented used oblique rotations given theoretical assumptions that neuropsychological processes are related, which was supported by the component correlation matrix data. Following suggested guidelines in establishing the relative importance of a variable to a given factor, only factor loadings with an absolute value greater than .4 were interpreted (as indicated by values denoted in bold in Tables 2 and 3; see Field, 2009). Next, composite scores for each of the neurocognitive domains obtained from these factor analyses were respectively formed over three steps: (1) individual raw subtest scores for each test were converted to z-scores; (2) these standardized test scores for each of the respective cognitive factors were then summed to create a domain score; and (3) composite scores for each domain were finally formed by converting the domain score into a standardized score via z-score transformation. The last phase employed hierarchal multiple regressions to investigate the contribution of demographic and neuropsychological factors to variance in step time at Year One and Two.

**Table 2:** Results of the Principal Components Factor Analysis at Year One.

Variables	Language/Verbal Memory	Executive Attention/Processing Speed	Working Memory
% of Variance	34.93	12.78	11.03
Logical Memory II	**.890**	−.052	−.006
Logical Memory I	**.882**	−.071	.008
VF Animals	**.599**	.016	−.002
VF Vegetables	**.572**	.199	.064
Boston Naming Test	**.479**	.247	−.048
TMT Trail A (secs)	.104	−**.921**	.097
TMT Trail B (secs)	−.057	−**.754**	−.147
Digit Symbol	.189	**.736**	−.045
Digit Span - Forward	−.176	.097	**.851**
Digit Span - Backward	.080	−.011	**.816**
Clocking Drawing Test	.261	−.232	.366

Notes: Bold values denote relevant coefficient loadings; Promax rotations with Kaiser normalization converged in 5 iterations. Trails Making Test (TMT) in seconds (secs); Verbal Fluency (VF).

**Table 3:** Results of the Principal Components Factor Analysis at Year Two.

Variables	Language/ExecutiveAttention/Speed	Verbal Memory	Working Memory
% of Variance	35.88	12.43	11.24
TMT Trail A (secs)	−**.828**	.165	−.034
Digit Symbol	**.745**	.095	−.030
TMT Trail B (secs)	−**.718**	−.043	−.132
Boston Naming Test	**.584**	.138	−.250
VF Animals	**.524**	.299	−.015
Clocking Drawing Test	.463	−.266	.247
VF Vegetables	.344	.292	−.030
Logical Memory I	−.045	**.944**	.078
Logical Memory II	−.001	**.943**	.024
Digit Span - Forward	−.032	−.008	**.883**
Digit Span - Backward	.010	.151	**.800**

Notes: Bold values denote relevant coefficient loadings; Promax rotations with Kaiser normalization converged in 5 iterations. Trails Making Test (TMT) in seconds (secs); Verbal Fluency (VF).

## Results

Neuropsychological test scores from 440 elderly individuals deemed cognitively normal at Year One were analyzed. 34 of these 440 individuals neuropsychological test performance significantly declined at Year Two. One-Way ANOVAs revealed that those that displayed cognitive decline (defined by a UDS score of 3 at Year Two) significantly differed from non-decliners at both Year One and Two on all neuropsychological subtests (*p*s<.05), but did not differ in MMSE scores, *p*s>.10. No significant group differences were found in sex or education. However, the cognitive decline group had a significantly higher mean age (*M* = 73.29, *SD* = 7.37) than the non-decliners (*M* = 70.28, *SD* = 6.91), *F* (1, 438) = 5.92, *p* = .015.

Repeated measure t-tests examined changes in neuropsychological test scores and step time from Year One and Two within each group. Table 4 presents the descriptive statistics (mean: *M* and standard deviation: *SD*) along with the *p*-values for the t-tests results. Within the non-decliner group, there was significant improvement on several of the neuropsychological test measures of memory and executive attention/processing speed. Conversely, the decliner group had significantly worse memory, executive attention/processing speed, and verbal fluency performance. As expected there were significant differences between step time in single as compared to dual task conditions at both time points within each group, *p*s<.001. No significant group differences were found in average step time for either conditions (*p*s>.05).

**Table 4:** Neuropsychological Characteristics at Year One and Two by UDS Groups.

Neuropsychological Tests	Year One		Year Two		
Non-Decliners (n = 406)	*M* =	*SD* =	*M* =	*SD* =	*p-*values
MMSE	29.25	1.09	29.29	1.07	0.534
Logical Memory I	14.58	3.07	15.07	3.41	0.001**
Digit Span - Forward	9.16	1.78	9.33	1.81	0.026*
Digit Span - Backward	6.93	2.14	7.24	2.04	0.001**
Verbal Fluency - Animals	22.07	5.27	21.85	5.44	0.263
Verbal Fluency - Vegetables	16.03	4.36	16.20	5.25	0.493
TMT Trail A (secs)	31.67	10.58	30.55	10.43	0.016*
TMT Trail B (secs)	76.41	28.44	75.44	28.93	0.407
Digit Symbol	50.64	10.48	51.34	10.82	0.005**
Logical Memory II	13.90	3.26	14.48	3.56	0.00**
Boston Naming Test	28.44	1.79	28.53	2.19	0.354
Clocking Drawing Test	16.95	1.35	16.84	1.47	0.181
Dual Task Step Time	.5712	.063	.5675	.0578	0.125
Single Task Step Time	.5405	.0500	.5388	.0443	0.288
**Cognitive Decliners (n = 34)**					
MMSE	28.50	1.31	28.59	1.52	0.731
Logical Memory I	12.18	2.77	11.06	4.10	0.042*
Digit Span - Forward	8.88	1.61	8.21	2.33	0.033*
Digit Span - Backward	5.74	1.94	6.26	2.18	0.113
Verbal Fluency - Animals	18.56	3.81	17.24	5.82	0.066†
Verbal Fluency - Vegetables	14.44	4.33	12.88	3.44	0.023*
TMT Trail A (secs)	35.82	8.45	41.47	13.73	0.01**
TMT Trail B (secs)	95.52	39.83	116.39	68.07	0.004**
Digit Symbol	42.59	10.16	42.38	9.43	0.826
Logical Memory II	11.29	2.74	9.91	3.98	0.012*
Boston Naming Test	26.94	2.69	26.82	3.02	0.673
Clocking Drawing Test	16.38	1.76	15.68	3.00	0.092†
Dual Task Step Time	.5661	.0608	.5591	.063	0.414
Single Task Step Time	.5366	.0475	.5275	.0445	0.180

Notes: Mini Mental State Examination (MMSE); Trails Making Test (TMT) in seconds (secs).

† *p*<.10; * *p*<.05; ** *p*<.01

Neuropsychological test scores were submitted to principal components analysis at both time points. The loading coefficients of the neuropsychological tests on these factors at Year One and Two are respectively presented in Tables 2 and 3. Results at Year One provided a three-factor solution: Language/Verbal Memory, Executive Attention/Processing Speed, and Working Memory - that in combination explained approximately 59% of the variance. Year Two's test scores also generated a three-factor solution, which in combination accounted for approximately 60% of the variance. Similar to Year One findings, the digit span tests loaded on their own distinct “Working Memory” factor; however, Language measures (BNT and Verbal Fluency) loaded with Executive Attention/Processing Speed measures rather than on the “Verbal Memory” factor. Of interest, post-hoc split file analysis suggested that the pattern of coefficient loadings were largely similar across groups, however the magnitude of the relationship between spatial and verbal fluency measures with executive attention processing appeared to be stronger within the cognitive decliner group. However, given the small sample size for the cognitive decliners, these results will need to be replicated to increase confidence in their reliability. CDT displayed weak loadings with the other neurocognitive tests at both time points. Given the different factor loadings for language measures between the two time points, in order to allow for comparison, language was retained on its own factor in subsequent regression analyses.

Hierarchal multiple regressions were performed in order to quantify the unique contributions that demographic variables, body mass index (BMI) and neurocognitive functioning made to the dependent variable dual task step time. For each time point within each group, dual task step time served as the dependent variable, while demographic factors (age, education, and sex), BMI and neuropsychological factors (language, executive attention/processing speed, working memory, and verbal memory) served as the independent variables. Demographic variables and BMI were first entered into the model, followed by neuropsychological factors in the second step (Model Two). Table 5 presents the summary of these results within each group. In order to assess the unique contribution of predictor variables to the variance of the dependent measures, standardized beta coefficients (β) are provided for the variables that were statistically significant within Models One and Two for each group. Within the non-decliners, age and sex were significant predictors within the first model, however only sex (β = .256) and executive attention/processing speed (β = −.213) were significant in the final model once all of the factors were entered into the model. The final model accounted for 14.8% of the variance in step time. Similarly at Year Two, only sex (β = .287) and executive attention/processing speed (β = −.292) were significant in the final model, which accounted for 20.9% of the variance in step time in the non-decliner group. In the decliner group, neither model One or Two were significant at Year One; whereas at Year Two, the final model with all factors entered accounted for 50.4% of the variance in step time. Notably, only sex (β = .104) and executive attention/processing speed (β = .104) were significant within the final model, *F* (8, 25) = 3.17 *p*<.05.

**Table 5:** Contribution of Demographic and Neuropsychological Factors to Dual Task Step Time by Group and Year.

Non-Decliners (*n* = 367)	Year One			Year Two		
	*Adj. R^2^*	Δ*R^2^*	ΔF	*Adj. R^2^*	Δ*R^2^*	ΔF
**Model 1:** BMI and Demographic Factors	*0.112*	0.120	13.73**	*0.152*	0.161	19.18**
**Model 2:** Neuropsychological Factors	0.131	0.028	3.24**	0.192	0.048	5.97**

Decliners (*n* = 30)	Year One			Year Two		
	*Adj. R^2^*	Δ*R^2^*	ΔF	*Adj. R^2^*	Δ*R^2^*	ΔF
**Model 1**: BMI and Demographic Factors	0.100	0.209	1.92	0.036	0.153	1.31
**Model 2**: Neuropsychological Factors	0.165	0.158	1.57	0.345	0.351	4.42**

Notes: Body Mass Index: BMI; Adjusted: Adj.; *p*-values: ** *p*<.01.

## Discussion

The present study examined neuropsychological characteristics that appear to be the most sensitive to change in the elderly, and the influence of these characteristics along with demographic factors on gait step time at two distinct time points. Consistent with our hypothesis that the neuropsychological test battery would be sensitive to detect mild changes in cognition within the elderly, a subset of participants displayed decrements in their memory, executive attention, and verbal fluency performance; however, impairments were not noted on all of the tests (e.g., digit coding and BNT) that compromised these neuropsychological domains. Here, it is important to note the lack of sensitivity of the MMSE, which failed to detect mild changes in cognition. Interestingly, improved working memory, memory, and executive attention/processing speed performance was found on several of the test measures within the majority of participants at Year Two. Contrary to our hypotheses, a three – and not four factor solution best fit the neuropsychological data. Additionally, while these three factor solutions were relatively stable across the two time points, the language measures loaded on different neuropsychological domains. These results taken together, suggest that there is some variance in the factor structure of the UDS battery when repeated overtime. Implications of these findings are discussed in detail below.

As expected, neurocognitive factors predicted variance in dual task step time at both time points even after controlling for demographic factors. Notably, the contribution of Executive Attention/Processing Speed to gait variance substantially increased at Year 2 for the “cognitive-decline” group.

Although not part of original hypotheses, *practice effects* were found by comparing data collected at Year One to those collected at Year Two using repeated measure *t*-tests. Improved performance was observed within the non-cognitive decline group on measures of working memory, verbal memory, and processing speed at Year Two. Importantly, practice effects, which are believed to constitute the acquisition of test taking skills due to prior test exposure, have been previously found on tests of memory, working memory, and/or that have a strong motor component to them (see [32]). Consistent with past findings, WMS-R Logical Memory and digit span tests appeared to be affected by the previous test exposure a year prior, while performance enhancement at Year Two was not found for the verbal/language ability tests in the non-decliners (see [33], [34]). Of clinical relevance, a subset of participants (the decliners) failed to exhibit performance enhancement and instead demonstrated a performance decrement on these test measures at Year Two. There is research to suggest that failure to benefit from previous test exposure on tests that are susceptible to practice effects may be an indicator of a decline in that cognitive process [35], and that failure to exhibit expected practice effects on certain neuropsychological tests substantially increases with age [34], [36]. In conclusion, these results support the notion that failure to exhibit practice effects may be an important indicator of age-related cognitive decline. In this regard, examination of test-retest stability may be useful in prognosis of MCI.

Neuropsychological test scores were submitted to factor analysis at two time points spanned approximately a year apart. Based on prior research [24], we hypothesized that the neuropsychological test battery would provide a four-factor solution of: Attention/speed of processing, executive function, episodic memory, and language. However, at both Year One and Two, three factors that were capable of explaining approximately 60% of the variance in the neuropsychological test measures were extracted at each respective time point. Notably, the obtained factor solution at Year One differed from that of Year Two, in which language measures (BNT and Verbal Fluency tests) loaded with Verbal Memory at Year One and loaded with Executive Attention/Processing Speed at Year Two. The Year Two loading of language measures is consistent with research that suggests verbal fluency tasks also require executive control in both healthy controls as well as individuals with MCI [37]. All other factor loadings remained relatively stable across the two time points. Of further interest, the digit span tests did not load with the other neuropsychological tests considered to be proxies of attention (i.e., Digit Coding and TMT subtests). Importantly, while the UDS battery defines the digit span subtests as measures of attention, these subtests were designed primarily as measures of working memory [30]. Thus, it is not entirely surprising that these tests loaded separately from the executive attention/processing speed measures, which also have a large psychomotor component to them. Overall, these results indicate that these neurocognitive factor structures are relatively stable. It is possible that the differential loading of language measures on Verbal Memory at Year One and Executive Attention/Processing Speed at Year Two was a result of the fact that language measures typically do not appear to be susceptible to practice effects, whereas memory tests tend to show large practice effects.

Consistent with past research, regression analyses revealed that both Executive Attention/Processing Speed and Sex predicted variance in dual task step time at both time points within each group. Notably, the contribution of Executive Attention/Processing Speed to gait variance substantially increased at Year Two for the cognitive decline group. These findings are in line with previous research that has found poor attention allocation is related to greater gait variance in those with MCI, and that has posited that decline in executive attention underlies the relationship between age-related decline and decrements in dual-task performance. Gender also explained a substantial amount of variance in walking gait, in which females had faster step times than males. Unfortunately, there is a paucity of research that has examined putative underlying sex differences in neuropsychological abilities (e.g., spatial vs. language abilities) in relation to gait patterns. Thus, we suggest that investigation of potential sex differences in underlying cognitive processes in relationship to changes in gait patterns and risk of falling warrants future research.

A limitation of this study is that participation was limited to enrollment in LABrainS, which may affect the generalizability of these findings. LABrainS participants tend to be college educated, predominantly white, with a higher proportion of females than males. Thus, extending these findings to a broader demographic will be an important future direction. The present study was also limited by the fact that such a small percentage of the sample were “decliners”, although it should be pointed out that despite the relatively small sample of “decliners” we were able to make significant findings in terms of cognition-gait interactions.

In summary, we have replicated research that suggests that executive attention/processing speed plays an important role in gait variance. We have also added to this literature in that this relationship appears to be dynamic in that the magnitude of contribution increased across time in those who displayed cognitive decline at Year Two. Additionally, we found that a three and not a four factor structure best explained the UDS neuropsychological test battery; and, overall these factor structures were relatively stable with the exception of language measures. Lastly, while more research is needed within this area, it appears that the failure to learn testing material and/or to learn how to approach the task more effectively with repeated administrations of the tests (particularly, attention/processing speed or memory tests) might prove to be useful of indicator early dementia. Future research will need to address whether these factors (e.g., increased step time variability in response to cognitive distraction) are also linked to heightened risk of falls within the elderly and the degree to which they are able to predict dementia within the elderly.
